# Healthcare utilization and disease burden in children with metachromatic leukodystrophy in Germany

**DOI:** 10.1186/s13023-025-03637-z

**Published:** 2025-05-23

**Authors:** Christiane Kehrer, Andrea Bevot, Pascal Martin, Christa Raabe, Saskia Gregor, Ingeborg Krägeloh-Mann, Samuel Groeschel

**Affiliations:** 1 Department of Paediatric Neurology and Developmental Medicine, University Children’S Hospital, Hoppe-Seyler-Straße 1, 72076 Tübingen, Germany; 2 Department of Neurology and Epileptology, Hertie Institute for Clinical Brain Research, University of Tuebingen, 72076 Tübingen, Germany

**Keywords:** Metachromatic leukodystrophy, Neurodegenerative disease, Disease burden, Healthcare utilization, Diagnostic pathway, Sociomedical aspects, Caregivers’ interviews

## Abstract

**Background:**

Metachromatic leukodystrophy (MLD) is a life-limiting neurodegenerative disease due to pathogenic variants in the *ARSA* gene. Patients experience severe neurological symptoms, developmental regression, and early death. Aim of the study was to analyze disease burden and healthcare utilization in different stages of the disease in children with late infantile and juvenile MLD in Germany.

**Methods:**

Out of a nationwide total cohort study (TC) (n = 83), we undertook telephone interviews in a representative follow-up cohort (FC) defined by advanced disease stages (n = 19). The FC allowed detailed long-term data of the disease in addition to cross-sectional data of the TC.

**Results:**

Nearly all patients showed spasticity, truncal hypotonia and dysphagia, and about half of the patients developed epilepsy. Most children required special education; none finished regular school. Analysis of the FC showed that neuronal intestinal burden was extensive, including obstipation (57%), micturition problems (47%), and tube feeding (63%). Gallbladder polyposis was seen in 52%. General well-being did not strongly correlate with motor function, whereas pain was associated with reduced well-being. Baclofen, Omeprazole, Vigabatrin and Polyethyleneglycol were the most frequently used drugs. Patients took up to 15 different drugs daily. Altogether, 127 hospitalisations (485 treatment days) were registered in the FC (median age 9 years, median one inpatient stay per patient per year). Diagnostic procedures were main reasons for hospitalization (29 hospitalizations, 128 treatment days), and accounted for the main burden for families (68%). The median use of 15 different devices (maximum 29) throughout life illustrated a high burden of the disease. During disease course, there was a change from more “active” devices (e.g., walker) to more “passive” devices (e.g., form seat). Physical therapy was the most relevant therapy in advanced disease stages (100%), while occupational therapy or speech therapy primarily were used in early disease stages. State welfare benefits, home- and palliative care were used broadly.

**Conclusion:**

Diagnostic and treatment routine pathways and sociomedical support in MLD require extensive resources. We provide detailed cross-sectional and long-term data of MLD-associated disease burden in different stages of disease. This data may serve as a reference when analyzing disease- and healthcare burden also after gene-/stem cell-therapy.

**Supplementary Information:**

The online version contains supplementary material available at 10.1186/s13023-025-03637-z.

## Introduction

Metachromatic leukodystrophy (MLD) is an orphan genetic, neurodegenerative, and life-shortening disease due to pathogenic variants in the *ARSA* gene*.* Deficiency of the encoded enzyme arylsulfatase A (ARSA) leads to increased storage of sulfatides in different tissues and causes widespread demyelination of the central and peripheral nerve system [1, 2]. Affected patients suffer from multiple neurological symptoms, and experience regression of motor, cognitive and behavioral function, and early death [1–6]. The natural disease course is influenced by age of onset [1–6] as well as type of symptoms at onset [5, 6].

Haematopoietic stem-cell transplantation (HSCT) is recommended for MLD patients with a late-onset form of the disease in a pre-symptomatic or early-symptomatic disease stage [7–12]. Gene therapy studies show encouraging results [13–15], also in long-term observation [15]. The ex-vivo autologous lentiviral gene therapy product Libmeldy® for treatment of early-onset MLD has been approved in Europe since 2020 [16]. Upcoming therapies and pending newborn screening [17–19] for life-shortening MLD require detailed reflection of the disease burden.

Leukodystrophies are burdensome and raise relevant healthcare costs [20–23], even after HSCT [24, 25]. Some studies report data on costs of diagnostic procedures and routine care in overall leukodystrophies [20–24, 26], but not exclusively in MLD. There are few studies targeting disease burden specifically in MLD, for example with focus on hospitalization and clinical outcome [27–29] or on aspects of quality of family life [30, 31]. Within the scope of an international survey, Sevin et al. described disease burden in MLD from the point of view of caregivers [31] and Thomas et al reported on current results of a caregiver survey concerning disease burden in MLD in the UK and Republic of Ireland [32].

In this study, we analyze healthcare utilization and disease burden in different stages of disease in children with MLD in Germany with respect to diagnostic and routine procedures, clinical symptoms, drugs, inpatient and outpatient care, devices, supportive therapies, education, and sociomedical- and family aspects. These data may serve as reference when evaluating outcome after gene-/stem cell-therapy and may therefore be helpful for interpretation of impact on the healthcare system.

## Methods

*Patients recruiting:* Patients were recruited since 2005 within the nationwide scope of the German research network LEUKONET. Informed consent was given by the parents in all cases. The study (DRKS 00030287) was approved by the Ethical Committee of the University of Tübingen (Nr. 401/2005).

*Diagnosis:* Diagnosis of MLD relied on clinical, enzymatic (ARSA deficiency) and genetic findings, confirmed by cerebral magnetic resonance imaging (cMRI) and increased urinary sulfatides.

*Definition of MLD forms:* Forms of MLD were divided into a late-infantile form (li) (onset of < / = 30 months), an early-juvenile (EJ) (onset of > 30 months < 6 years) and a late-juvenile form (LJ) (onset = / > 6 years < 16 years) according to the literature [1–6, 28].

*Definition of distinctive disease stages:* To describe disease progression, we introduced 3 disease stages related to gross motor function assessed by the Gross Motor function in MLD (GMFC-MLD) [33] as follows: stage 1: GMFC-MLD 0–1 (able to walk freely with or without quality normal for age); stage 2: GMFC-MLD 2–4 (no more able to walk freely but some locomotion OR/AND sitting); stage 3: GMFC-MLD 5–6 (loss of any locomotion with or without head control preserved). This was done analogously to Fumagalli et al. [6].

*Study design:* In this national, non-interventional cohort study we describe cross-sectional and long-time data of MLD-related disease burden and healthcare utilization in different stages of the disease under defined aspects (see below). Cross-sectional data were obtained from a total cohort (TC) by parental questionnaires and medical records at date of questionnaire (DQ). Long-time data were obtained from a follow-up cohort (FC), consisting of children in their advanced disease stage, by standardized telephone interviews with parents according to established procedures [27, 28, 31].

Healthcare and disease burden aspects comprised diagnostic and routine treatment procedures, clinical symptoms, presence of pain, drugs, inpatient- and outpatient care, devices, supportive therapies, education, and, additional for the FC, socio-medical and family aspects. 

### Overall total cohort

*Definition*: As this study focuses on healthcare burden in children with MLD in Germany, patients were excluded from the German LEUKONET cohort (informed consent n = 128) under following conditions: adult onset, family lives abroad, or coincidence of another neurological disease. The remaining patients were summed up to the total cohort (TC) (n = 83).

### Follow-up-cohort

*Definition*: To define a cohort in which healthcare utilization reflects current standard and average family need in progressed disease stage, we defined additional inclusion/exclusion criteria for follow-up analysis: patients only in advanced disease stage 3 (GMFC-MLD 5–6), diagnosis within the last 10 years, genetic confirmation, age < 18 years at date of interview (DI), death less than 5 years ago at DI, no affected siblings, no study patients (intrathecal enzyme replacement therapy (ERT) or gene therapy), no language barriers hampering telephone interview. (We didn’t involve families with several affected children, as data of number of devices (used crosswise among siblings), or times for parental leaves etc. might not be comparable to families with only one affected child). These patients were summed up to the follow-up cohort (FC) (n = 19).

*Telephone interviews:* Telephone interviews with parents (14 mother, 4 father, 4 both parents) were scheduled for 60–90 min (CK). 54 defined device products and 18 defined supportive therapies were listed for the interviews with the possibility to name further, non-listed devices (“others”).

Age at interview or at death (n = 4) ranged from 2.7 to 18 years (median 9 years) (Tables 1, 2).

**Table 1:** Baseline data of the total cohort (n = 83)

Total cohort: 83 patients (34 male, 49 female)	39 late-infantile	21 early-juvenile	23 late-juvenile
Treatment (total n = 23)	3 ERT i.v., 1 HSCT	9 HSCT	10 HSCT
Death (total n = 38)	19	11	8
Foreign ancestry (total n = 18)	14	1	3
Type of symptoms at disease onset	34 motor	12 motor	2 motor
	4 motor and cognitive	6 motor and cognitive	8 motor and cognitive
	0 cognitive	0 cognitive	10 cognitive
	1 presymptomatic	3 presymptomatic	3 presymptomatic
Disease stage *(data of questionnaire)*			
Stage 1 (GMFC-MLD 0 or 1)	3	7	15
Stage 2 (GMFC-MLD 2–4)	10	3	1
Stage 3 (GMFC-MLD 5 or 6)	26	11	7
Time from disease onset to entry into stage 2 [months]	0–23; median 4.9	5–120; median 21.0	3–116; median 28.9
Time from disease onset to entry into stage 3 [months]	4–88; median 13.0	6–92; median 24.5	5–67; median 28.0

**Table 2:** Baseline data of the follow-up cohort (n = 19)

Follow-up cohort: 19 patients (9 male, 10 female), all disease stage 3	11 late-infantile	4 early-juvenile	4 late-juvenile
Treatment (HSCT) (total = 5)	None	3	2
Age at HSCT [years]	–	5.1–7.7; median 5.7	11.7–13.5; median 12.6
Dead at time of interview	2	1	None
Type of symptoms at disease onset	10 motor 1 motor and cognitive	3 motor 1 motor and cognitive	3 cognitive 1 motor and cognitive
Age of onset [months]	14–24 (median 20)	32–63 (median 54)	82–135 (median 107)
Age of diagnosis [months]	23–37 (median 27)	40–92 (median 62)	92–161 (median 145)
Interview age [years] (retrospective observation time)	2.7–11.5 (median 6.0)	9.8–11.7 (median 11.8)	11.6–20.0 (median 18.6)
Time from disease onset to entry into stage 2 [months]	0–23; median 4.3	9–44; median 14.0	24–64; median 32.0
Time from disease onset to entry into stage 3 [months]	5–34; median 14.3	12; (single value)	28–56; median 42.0

**Fig. 1:**
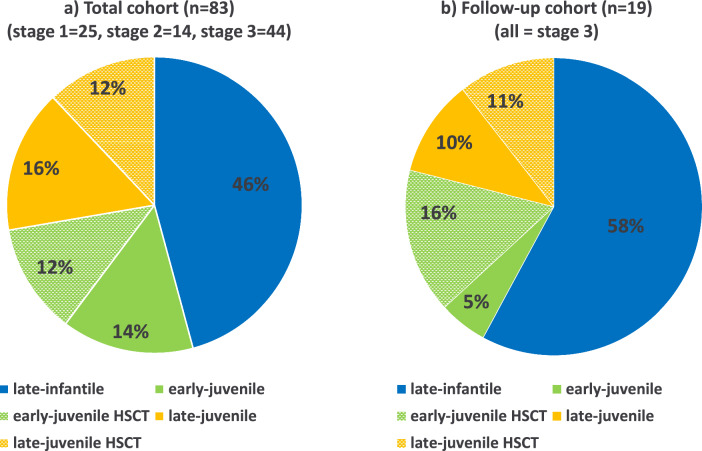
Distribution of MLD forms and treatment (HSCT) in the **a** total cohort and **b** follow-up cohort

Concerning the distribution of MLD forms and the distribution of natural history compared to HSCT, the FC (n = 19) consistently represented the TC (n = 83) (Fig. 1).

## Results

### Total cohort (TC) (n = 83) (Table 1)

Demographic data of the total cohort are shown in Table 1 as distribution of sex (34 male; 49 female), presence of foreign ancestry, and MLD form (39 LI patients; 21 EJ patients; 23 LJ patients). It shows the distribution of former treatment (i.v. ERT; HSCT), the type of onset symptoms (motor/motor and cognitive/cognitive/presymptomatic) and the number of deceased patients (19 LI; 11 EJ; 8 LJ) per MLD form. Disease stage at DQ (date of questionnaire) is also shown in Table 1 (25 patients in stage 1; 14 in stage 2; 44 in stage 3), as well as time from disease onset to entry into stage 2 and stage 3 per MLD form. Age at DQ ranged from 0.8 to 17.4 years (median 7 years). The lifespan of the patients who had already died at DQ was 2.4 to 28.3 years (median 13 years).

### Follow-up cohort (FC) (all stage 3) (n = 19) (Table 2)

Demographic data of the follow-up cohort are shown in Table 2 as distribution of sex (9 male; 10 female) and MLD form (11 LI patients; 4 EJ patients; 4 LJ patients). It shows the distribution and age of HSCT (3 EJ patients; 2 LJ patients), the type of onset symptoms (motor/motor and cognitive/cognitive/presymptomatic) and the number of deceased patients (2 LI; 1 EJ; 0 LJ) per MLD form. Information is given about the age of onset (months), age at diagnosis (months) and age at interview (years) per MLD form (median interview age: 6 years (LI); 11.8 years (EJ); 18.6 years (LJ)). Table 2 also shows the time from disease onset to entry into stage 2 and stage 3 per MLD form.

### Diagnostic procedures

ARSA deficiency was determined by assay in leukocytes in all patients (TC n = 83; FC n = 19). CMRI was done in all patients of the FC and nearly all (n = 82) of the TC. Molecular genetic analysis was also done in all patients of the FC and in most of the TC (n = 62, 75%). This was followed by examination of urinary sulfatides (TC: n = 67 patients (81%); FC: n = 17 patients (89%)), nerve conduction velocity (NCV) (TC: n = 59 patients (71%); FC: 17 patients (89%)) and lumbar puncture (TC: n = 37 patients (44%); FC: 7 patients (37%)). EEG was done in one patient of the FC (5%) and was not documented in the TC.

### Neurological and clinical symptoms

**Fig. 2:**
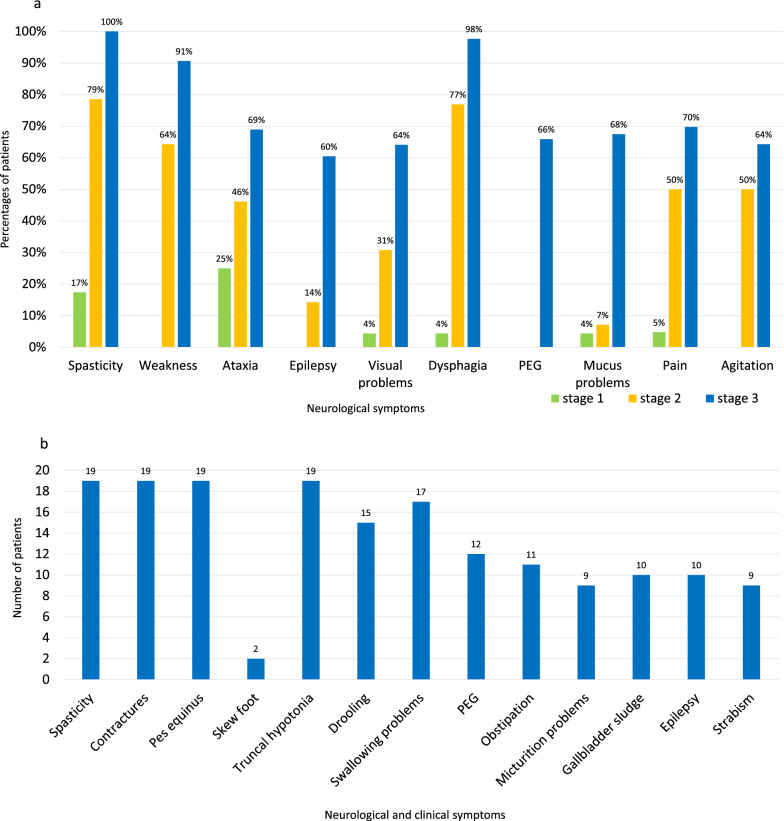
**a** Neurological symptoms in the total cohort (n = 83) per disease stage. (Y axis: Percent of patients; X axis: Neurological symptom). (“PEG” means “percutaneous endoscopic gastro-tube”) (Stages are defined as follows: Stage 1: GMFC-MLD 0–1 (able to walk freely with or without quality normal for age); stage 2: GMFC-MLD 2–4 (no more able to walk freely but some locomotion OR/AND sitting); stage 3: GMFC-MLD 5–6. (loss of any locomotion with or without head control preserved). **b** Neurological and clinical symptoms in the follow-up cohort (n = 19). (Y axis: Number of patients; X axis: Neurological and clinical symptom)

Distribution of neurological symptoms in the TC (spasticity, weakness, ataxia, epilepsy, visual problems, dysphagia, need of percutaneous endoscopic gastro-tube (PEG), mucus problems, pain, agitation) per disease stage is shown in Fig. 2a (given in percentages of patients). Each item was observed most frequently in stage 3, followed by stage 2 and less often in stage 1. Spasticity, dysphagia, and weakness were the most frequent symptoms in general, occurring in most patients in stage 3 (90–100%) and were still frequent in stage 2 (64–77%). In stage 1, some symptoms were only very rare, and weakness, epilepsy and agitation did not occur.

Distribution of neurological and clinical symptoms in the FC (spasticity, contractures, pes equines, skew foot, truncal hypotonia, drooling, swallowing problems, PEG, obstipation, micturition problems, gallbladder sludge, epilepsy, strabismus) is shown in Fig. 2b (because of the smaller cohort given in number of patients). Swallowing problems occurred at the age of 2–17 years (median 2.8), epilepsy at the age of 5–17 years (median 6) and PEG was necessary at the age of 2–10 years (median 3). 10 patients were completely and 2 patients partly tube-fed, 9 had special nutrition; 9 patients reported PEG-associated problems.

16 patients (84%) regularly underwent ultrasound of the gallbladder, 7 (37%) took ursodeoxycholic acid against sludge, and 5 patients (26%) underwent cholecystectomy. 7 patients (37%) regularly underwent ultrasound of the urinary bladder, 9 (47%) did daily urinary bladder tapping, and 3 patients (16%) used daily catheterization. Additionally, there was the need of mucus suction in 10 patients (53%) (5 during infections, 3 regularly, 2 daily).

### Pain and well-being

In the FC, the closed-ended question of the parents’ form “how is your child’s general condition?” was answered with “good” in 11 cases (58%), with “moderate” and “reduced” in three cases each (16%), and “poor” in two cases (11%). “Pain” (regardless of its cause) was observed in 9 patients (47%); of these three patients each were reported to be in a “good” and “moderate” general condition, two patients in a “reduced” and one patient in a “poor” general condition. Pain during the disease course got “better” in 11 patients (58%), “worse” in four patients (21%) while there were “no tendency” in four patients (21%).

### Drugs

Drug use was higher in the advanced disease stage, as in the TC no patient in stage 1 took any regular drugs (only medication on demand), but in stage 2, one patient took an analgesic, two patients took gastrointestinal drugs, and some patients took dietary supplements. In contrast, the 44 patients in stage 3 took cumulatively 137 drugs: 26 patients took cumulatively 38 analgesics, 22 patients took 27 spasmolytics, 21 patients took 26 sedatives, 13 patients took 14 anticonvulsants, 15 patients took 21 gastrointestinal drugs, 2 patients took 3 dietary supplements, and 5 patients took 8 other drugs. In the FC, the 19 patients showed cumulatively 121 drug uses. Some single patients took up to 15 different medical drugs and dietary supplements per day (FC).

**Fig. 3:**
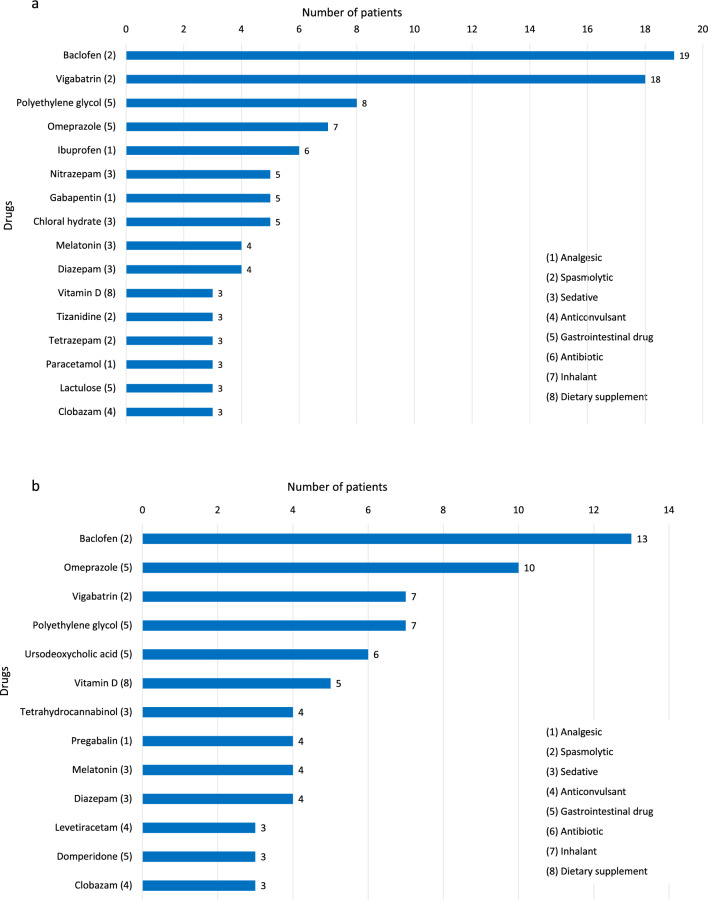
**a** Regular drugs in the total cohort (n = 83) (if used by more than two patients) (Y axis: Drug and drug group (indication they were used for); X axis: Number of patients using this drug). **b** Regular drugs in the follow-up cohort (n = 19) (if used by more than two patients) (Y axis: Drug and drug group (indication they were used for); X axis: Number of patients using this drug)

Drugs taken by more than two patients and their respective indication (drugs were grouped according to their indication) is shown in Fig. 3a for the TC and in Fig. 3b for the FC. (An overview of all drugs and the respective group is given in Supplementary Table 1a for the TC and in Supplementary Table 1b for the FC.)

With respect to drugs on demand, Midazolam (anticonvulsant) was used by 7 patients, Diazepam (sedative) by six, chloral hydrate (sedative) by four, and metamizole (analgesic) by 3 patients (FC).

### Inpatient stays and outpatient visits

**Fig. 4:**
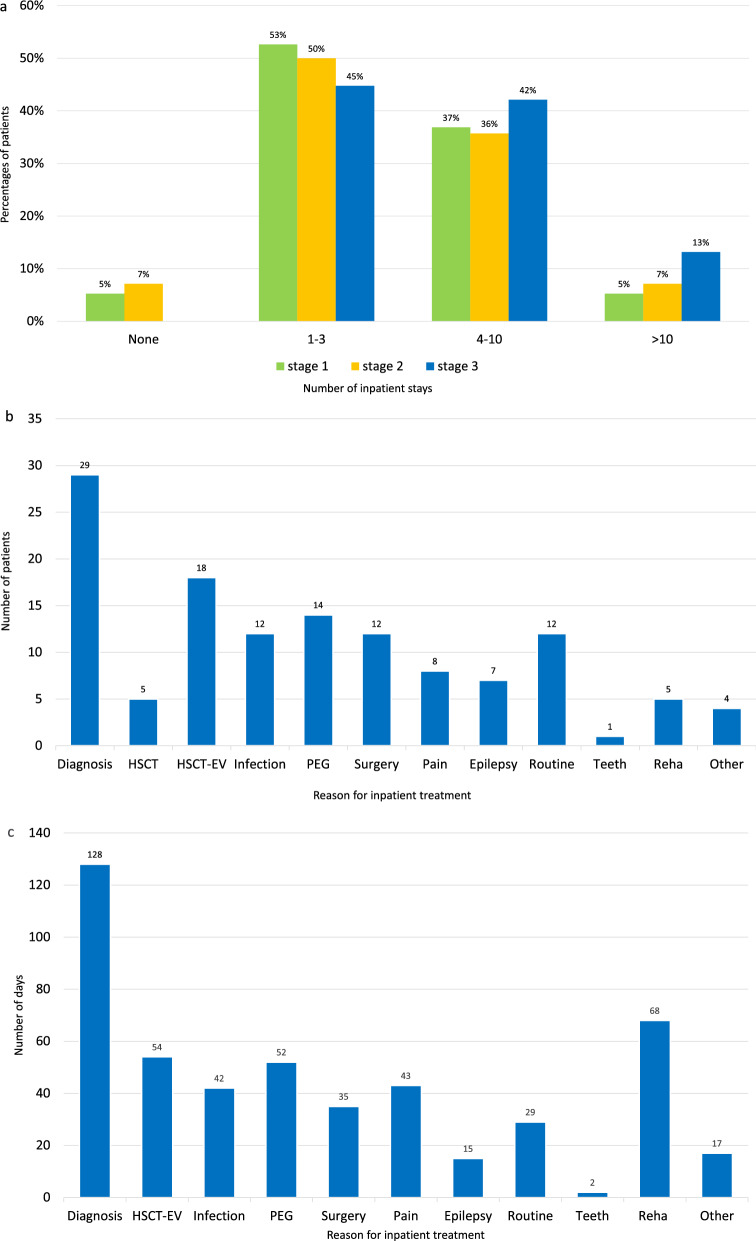
**a** Frequency (none/1–3/4–10/ > 10) of inpatient treatment in the total cohort (n = 83) per disease stage. (Y axis: Percentages of patients having inpatient treatment; X axis: Number of inpatient stays). (Stages are defined as above (Fig. 2a)). **b** Reason for inpatient treatment in the follow-up cohort (n = 19). (Y axis: Number of patients having inpatient treatment; X axis: Reason for inpatient treatment). (“HSCT” means “hematopoietic stem-cell transplantation”; “HSCT-EV” means “Evaluation after hematopoietic stem-cell transplantation”). **c** Duration (days) of inpatient treatment in the follow-up cohort (n = 19) (exclusively inpatient treatment for HSCT: 350 days in 5 patients)). (Y axis: Total number of days of inpatient treatment; X axis: Reason for inpatient treatment). (“HSCT-EV” means “Evaluation after hematopoietic stem-cell transplantation”)

The number of inpatient stays in the different diseases stages of the TC is shown in Fig. 4a. Patients in stage 3 tended to have more inpatient stays. Number of inpatient stays (cumulatively 127 stays) and distribution of the reason for inpatient stays (diagnostic procedures as most leading reason) in the FC is shown in Fig. 4b. Surgery beyond application of gastro-intestinal tubes concerned fundoplication, adductor tenotomy, and limb fracture surgery in one patient each (5%), and myo-fasciotomy, baclofen pump and dental surgery in two patients each (11%). Distribution of the duration of inpatient stays in the FC is shown in Fig. 4c. There were cumulatively 485 inpatient treatment days (including 68 days for rehabilitation but excluding 350 inpatient treatment days in 5 patients due to HSCT). Average duration of hospital stays (excluding rehabilitation stays) were reported in the FC within one week (range 1–14, median 5 days).

Total inpatient stays per patient (age at interview 9 years on average) in the FC reached up to 17 stays (median 7), and total outpatient visits per patient up to 24 (median 8). On average, there was one inpatient stay (maximum 10) and 3 outpatients visits (maximum 5) per patient per year (exclusively multiple infection controls by the local paediatrician in 2 patients within a defined time period). Only four patients (21%) were treated on intensive care. Concerning medical disciplines, 8 patients of the FC (42%) regularly saw their local paediatrician, (2 of them more than 10 times per year for infection control), 6 patients (31%) went to see an ophthalmologist, and 9 (47%) a dentist.

### Devices

**Fig. 5:**
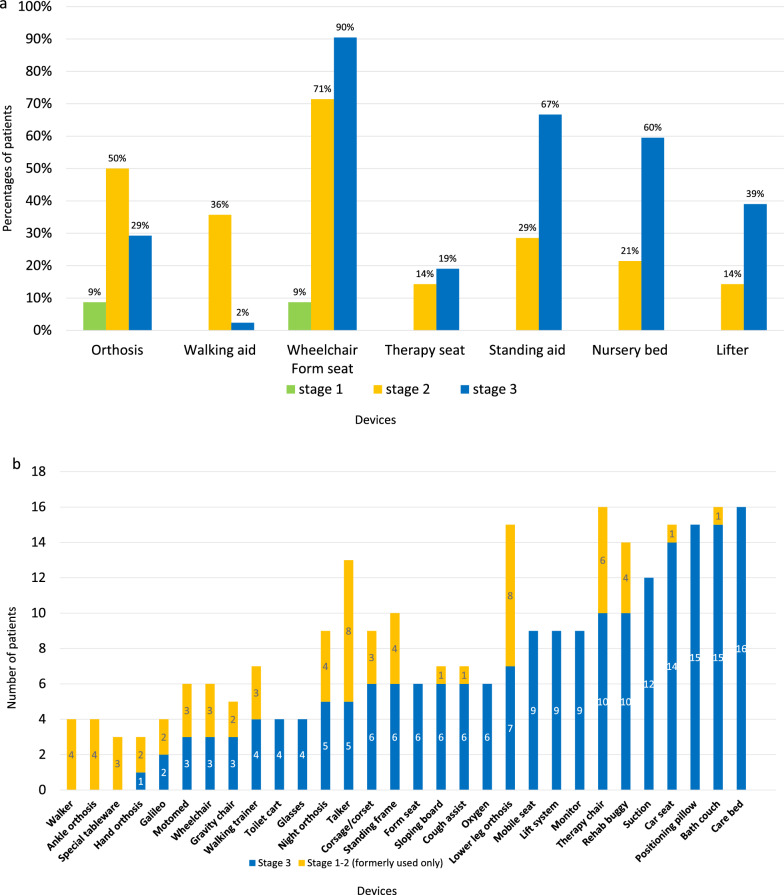
**a** Devices used per disease stage in patients of the total cohort (n = 83). (Y axis: Percentages of patients using the device; X axis: Device). (Stages are defined as above (Fig. 2a)). **b** Devices used per disease stage (stage 1, 2 formerly used only versus stage 3) in patients of the follow-up cohort (n = 19) (if used by more than two patients). (Y axis: Number of patients using the device; X axis: Device). (Stages are defined as above (Fig. 2a))

The use of individual devices per disease stage in the TC is shown in Fig. 5a (given in percentages of patients using the device (Y axis)). Wheelchairs and ridable form seats were the most frequently used devices; use increased with disease duration (9% in stage 1, 71% in stage 2, 90% in stage 3). Other “passive” devices were also primarily used in stage 3, less frequently in stage 2, and not observed in stage 1, whereas more “active” devices such as orthoses and walking aids were predominant in stage 2.

The use of 30 devices (if used by more than two patients) per disease stage in the FC is shown in Fig. 5b. As in the TC, there was a decrease in *active* devices (such as orthoses or walkers) and an increase in *passive* aids (for example devices for positioning such as a form seat or for vital intervention such as cough assist) over the course of the illness. Figure 5b shows, that the 19 patients (FC) used cumulatively 67 devices in illness stages 1 and 2 (20 different types of devices) and 196 devices in stage 3 (current situation) (27 different types of devices).

When looking at the use of assistive devices in all patients (without the restriction that at least two patients use it), an average use of 11 different assistive devices was reported as in use currently (range 5–23) and an average of 15 different assistive devices throughout the course of the disease (range 9–29).

### Supportive therapies

**Fig. 6:**
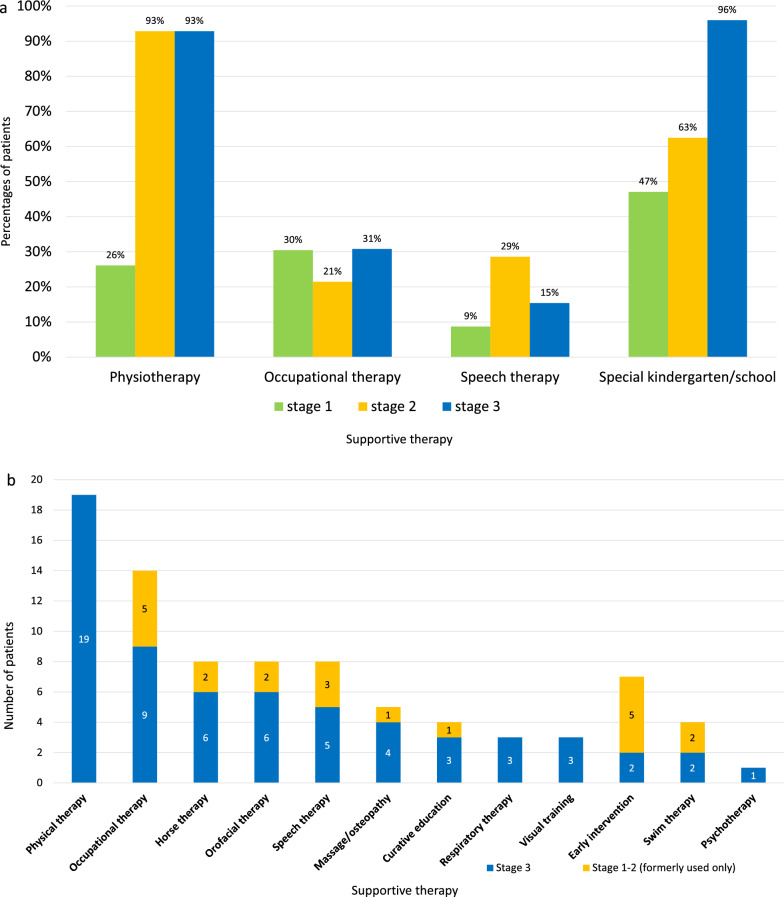
**a** Supportive therapies per disease stage in patients of the follow-up cohort (n = 83). (Y axis: Percentages of patients having respective supportive therapy; X axis: Therapy). **b** Supportive therapies per disease stage (stage 1, 2 formerly used only versus stage 3) in patients of the follow-up cohort (n = 19). (Y axis: Number of patients having respective therapy; X axis: Therapy). (Stages are defined as above (Fig. 2a))

Supportive therapies and need of special education in the TC per disease stage is shown in Fig. 6a. Physical therapy was used by over 90% of the patients in stage 2 and stage 3 and special schooling was reported in over 90% of patients in stage 3.

Supportive therapies per disease stage in the FC (stage 1, 2 vs. stage 3) is shown in Fig. 6b.

Physical therapy was used by all patients. In stage 1, 2 of the disease, occupational therapy, speech therapy and early intervention were the most frequent therapies.

Parents of the FC reported about 3 current supportive therapies on average (range 1–6), and a maximum to 5 (range 2–8) while entire disease course. Fourteen patients (74%) got therapy by home visit. Families spent 1–12 (median 5) hours per week for therapies.

### Education

In the FC, 2 children (11%) never went to any kindergarten/school and one child (5%) primarily went to a special-needs kindergarten. The other 16 children (84%) first went to a regular daycare/kindergarten (stage 1, 2); of these 16 children, 8 (50%) changed to a special-needs kindergarten during disease course, one child dropped out staying at home, two children (13%) went to special-needs school after regular kindergarten, and 5 children (31%) went to regular school after kindergarten (still stage 1–2) (1 EJ, 4 LJ). But no child could finish regular school. 8 patients (42%) had an assistant for kindergarten/school at any time, 11 children (58%) required special bus transport. Change of education system occurred at the age of 2–7 years (median 3).

### State welfare benefits and sociomedical aspects

In the FC, 14 families (74%) got support by palliative teams, 12 families (63%) by nurses, 9 families (47%) by a household help, and 9 families (47%) got other support. On maximum, 147 h per week to support care of the child (median 20) and 8 h for other support (median 1.5) were received.

All patients had an ID card certifying severe disability (“Schwerbehindertenausweis”), in all but one certifying the highest degree (“GdB 100”) together with all relevant disability codes (“Merkzeichen”). (Only one newly diagnosed child had a GdB of 70 at the beginning of the study). 6 patients (31%) were additionally labelled as “blind”. All patients received the highest care level (“Pflegegrad”). 18 families (95%) received full payment for respite care (“Verhinderungspflege”), and 6 families (31%) an additional care (“zusätzliche Betreuungsleistung”). The total costs paid by the families themselves or care and devices not covered by the healthcare system was reported by the parents as between 150 € and 100.000 € per family (median 5000 €). When parents were asked how long they have taken parental leave (beyond what had been planned before), they reported an additional period of up to 6 years (median 1.5).

### Participation in family life and family burden (only FC)

**Fig. 7:**
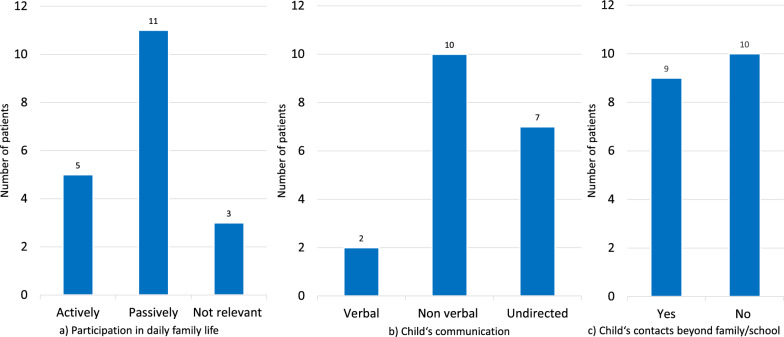
Psycho-social- and family aspects in the follow-up cohort (n = 19) as reported by the families. (Y axis: Number of patients; Y axis: Answer). **a** Participation in daily family life: “How does your child participate in daily family life?” **b** Child’s communication: “How does your child communicate?” **c** Child’s contacts beyond family and school: “Does your child have any contacts beyond family life and school?”

Patients’ communication skills, their participation in family life and their social contacts beyond family and school are shown in Fig. 7a–c.

“Fear of the future” was indicated by 14 families (74%) as one of the most burdensome items, “daily workload” by 6 families (31%), “seeing the child suffer” by 5 families (26%) and “nursing” by 4 families (21%). Thirteen families (68%) indicated the periods of “diagnosis” and “deterioration” as the most burdensome periods during the illness, whereas only one family referred to “HSCT” and “final stage” as such.

## Discussion

**MLD** is a neurodegenerative, life-shortening disease that not only causes severe suffering to the affected children but also places a great strain on families and the health care system. Data on MLD related burden and healthcare utilization are scarce [31, 32] or even lacking for some aspects of the daily life, for example concerning devices or supportive therapies. We provide detailed data on MLD-associated healthcare and disease burden focusing on the advanced disease stage on the basis of telephone interviews with caregivers of children in a late stage (FC), and within the context of cross-sectional questionnaire-data of an overall disease cohort from our German LEUKONET data base.

**Diagnostic procedures** accounted not only for the main reasons for hospitalization and most inpatient treatment days, but also for a major disease burden, as reported by the caregivers. The “period of diagnosis” was indicated by 68% of the families (FC) as the “most burdensome period of the disease”, which is remarkable in that the period of the "final stage", which is known to be very stressful, was practically not indicated as such. Diagnostic procedures included measurement of enzyme activity (ARSA) and sulfatide storage in urine, as well as molecular genetic testing and cMRI. These procedures are considered standard diagnostic procedures for MLD [34], cMRI being also important for decision on therapy. Other procedures, in part quite invasive, such as NCV or lumbar puncture, or poorly specific such as EEG, played a minor role. In fact, they are not essential for the diagnosis of MLD. In order to streamline diagnostic procedures in leukodystrophies, more invasive and poorly specific investigation tools could be omitted in the future.

In a former study, we could show, that time to diagnosis proved to be “long and distressing” (rating scale for parents: 9.3/10 scoring points) [30]. Median time between first symptoms and diagnosis in late-infantile MLD was shown to be around one year [4, 27, 29, 32] and often much longer for juvenile patients, ranging in different studies from nearly one year (11 patients) [29] to nearly two years (36 patients) [4]. It has been stressed, how important it is to pay attention to the first caregiver-reported signs to promote early diagnosis of MLD [29]. And the “diagnostic odyssey” in children with leukodystrophies has been discussed earlier [23]. These authors also showed that the costs concerning the period of diagnosis correlated with the number of diagnostic tests [23]. Thus, coming more quickly to a diagnosis will not only reduce the family burden, but also diagnosis-related costs. Shortening the time to diagnosis will also give higher chances when it concerns possible therapies [35].

With respect to **neurological symptoms**, spasticity, dysphagia, truncal hypotonia/weakness, and pain were the leading neurological symptoms in both MLD cohorts. About half of the patients developed epilepsy. The high prevalence of spasticity corresponds to what is reported in the literature [6, 27, 31, 32, 36, 37]. Of note is that seizures are reported with different prevalence in the literature, probably due to differences in sample sizes, age of the patients, stage of the disease, and observation period [31, 38].

Neuronal intestinal problems are known in leukodystrophies [6, 27, 28, 36, 37, 39–41] and are responsible for a relevant burden in daily life of the patients described in this study. MLD-specific gallbladder polyposis due to sulfatide storage was found in over half of the patients, consistent with literature data [32, 36]. In about one quarter of the patients, cholecystectomy was performed, and if not, most patients underwent regular gallbladder sonography as recommended in the literature [36, 40, 42]. Vital parameters remained stable over most of the lifetime. But two-thirds of the patients required regular mucus suction and over a quarter of patients had additional oxygen need, consistent with what is reported [27]. Respiratory failure has been described in late stages of LI MLD patients [32, 39]. Additional oxygen demand and abnormal vital parameters most likely mark a final stage of the disease.

Interestingly, deterioration or loss of motor function did not strongly correlate with reported reduced **general condition**. Further, absence of pain was more relevant in terms of overall satisfaction than motor ability was. Pain became less with disease progression in more than half of the patients (FC), but in another quarter of patients, it was reported as more severe with disease duration. Less pain might be due to use of analgesics, and/or receding neuropathic pain with increasing severity of neuropathy, but it is a limitation of this study that the exact causes of pain and its dynamics were not investigated in more detail. In our experience, pain must be considered as major burden of the disease. Pain is reported in the literature with variable frequency dependent on the stage and form of MLD [27, 28, 31]. So, management of MLD must focus on detecting the sources of pain and sufficient (analgetic) treatment. This concerns disease aspects such as obstipation, bowel obstruction, neuronal bladder dysfunction, gastroesophageal reflux, toothache, spasticity, joint dislocation, or bone fracture due to osteopenia, but also pain due to adverse drug effects [36, 39].

**Drug** use and burden due to medication were observed to increase with advancing disease stage**.** In general, spasmolytics and gastrointestinal drugs were the most frequently used drugs, followed by analgesics and sedatives. (*Note: Vigabatrin was classified as a spasmolytic since it was used because of its antispastic and not its antiepileptic effects)*. Van Haren et al. list Amitryptiline, Baclofen, Diazepam and Gabapentin (alphabetical listing) as “common symptomatic medication” against pain and spasticity in leukodystrophy patients [39]. This corresponds to our findings with the exception of Amitryptiline, which played no relevant role.

Concerning spasticity, intrathecal baclofen in MLD has been shown to be a”feasible therapy to improve daily care and comfort”, and authors underline that it should be considered early [43].

The high prevalence of gastrointestinal drugs in this study reflects the burden caused by neuropathic gastrointestinal problems discussed above. This is in line with what is reported and recommended in the literature [39], e.g. increased hydration, stool softener and laxatives (e. g. Polyethylene glycol) and the use of Omeprazole as an effective drug against gastroesophageal reflux [36]. A recent large study of a caregiver assessment in Pelizaeus-Merzbacher Disease (PMD) indicates the importance of these drugs, as 60% of the patients took vitamins and supplements, and 45% medication against constipation, which both were more frequent than antispastic drugs (39%) or anticonvulsants (14%) [41]. In contrast, authors of a caregiver survey of MLD in the UK/Ireland report muscle relaxants, pain medication and anti-secretion medication as more frequent (each 88%) than gastrointestinal drugs (63%) in ten LI patients, which could be an effect of the smaller group and younger age (median 5.6 years) [32].

Anticonvulsants belonged in our cohort to less frequently used drugs, as epilepsy, known as a late-onset symptom [39], occurred in about half of patients only. Of note, however, if epilepsy was manifest, more than one anticonvulsant was needed, indicating that epilepsy could be difficult to treat. In our FC, Clobazam, Levetiracetam and Phenobarbital were the most frequently used anticonvulsants, while Carbamazepine, Lamotrigine, and Valproic were less used. This is in contrast with what was mentioned in another survey [39]. Authors recommend the use of drugs “capable of concomitantly treating seizures along with other comorbid symptoms to avoid polypharmacy” [39]. In our cohort, this was true for Vigabatrin or Gabapentin, for example. The minor role of Botulinum-toxin injections and its relatively poor effect in this study may be due to advanced structural limb contractures in most patients. Some authors propose focal Botulinum toxin to improve hygiene (M. adductor) or ambulation (M. gastrocnemius) [36]. But, there is a recent report on the risk of inducing overall weakness as adverse drug effect in a LI child after gene therapy [16]. We think it is worth discussing the early use of botulinum toxin with the primary aim of pain prevention, reducing spasms, or improving manageability.

The burden of **hospitalization** is expressed by number and duration of inpatient stays as well as number of outpatient visits. Our data show on average about one inpatient stay per patient per year, which is similar to results of a recent survey on 98 patients with Krabbe disease over a period of seven years [25]. For MLD, two hospitalizations in the previous year (median) were reported in 31 MLD patients of an international survey [31], and 3 hospitalizations respectively for 8 LI MLD patients from UK/Ireland [32]. Duration of inpatient stays on average was about one week (excluding rehabilitation stays), similar to what has been reported by others [27, 31, 32]. Costs for the healthcare system concerning inpatient care of leukodystrophy patients in comparison to other care forms have been described in the literature [20–24].

The observation that the most common reason for an inpatient stay is related to the diagnosis was quite unexpected, since the patient group described here (FC) was diagnosed within the last 10 years. One could have expected that easier access to genetic diagnostics and the broader knowledge of MLD, which has received some attention in recent years, would significantly shorten the time to diagnosis. Although the aim remains to counsel families in detail, it also remains a goal to shorten inpatient stays for diagnosis.

A survey of Krabbe patients found that epilepsy treatment was among the most common reasons for hospital admission (along with failure to feed or thrive) [25]. This is in contrast to our data in MLD, in which hospital admissions due to epilepsy treatment were less common.

Number of annual outpatient hospital visits in our cohort (range up to 5) corresponds well to what was shown for 5 German MLD patients taking part in an international study, while there were much higher numbers of median outpatient visits in the previous 12 months for other countries (for example in the median 43 for France (range 2–200) [31] or 12 (range up to 40) [31] and 18 (range up to 100) [32] for the UK. The lower numbers for outpatient appointments in the German cohort may be related to the fact that in Germany this only refers to visits at the doctor’s (no therapist contacts or contacts with the palliative care team). Bonkowsky et al. described the important role of the local pediatrician in managing the case, mediating between parents and specialists, and in preventing complications [26]. In Germany, this may be the task of a social pediatric center (“SPZ”), while local pediatricians rather are responsible for vaccinations and treatment of infections as shown in our study. Some authors recommend that medical visits in leukodystrophy patients should be “scheduled at last every 6 months” to “reassess and reprioritize care needs” [39]. But others point out that frequent doctor contacts are stressful for parents and have an impact on the quality of life [26, 44].

The wide use of **devices** in our cohorts during the patients` disease, also illustrates the high burden of this disease. While in patients with genetically determined leukoencephalopathy, a “lower health-related quality of life” was shown in children “using a wheelchair” [45], the general “benefit from orthotics and mobility equipment” has been underlined [26]. In order to assist mobility, the use of devices such as orthoses, gait trainers, walkers, lifts, and standing frames in leukodystrophy patients is recommended in a revised consensus statement on care of leukodystrophy patients [36]. Consistent with the progressive nature of the disease course in MLD, some assistive devices are useful early in the disease course (e.g., walking frames), but no are longer used when disease progresses and will be replaced by others. Accordingly, we could show in both our cohorts that during the disease course there was a change from more “active” devices (such as orthoses, walker, walking trainer, active wheelchair, Motomed^R^, talker, etc.) to more “passive” devices. Passive devices included standing frames, toilet carts, lifts, and any ridable (mobile) seats, cushions for positioning the whole body in a prone or supine position, care beds, as well as devices supporting vital function (cough-assist, oxygen, suction). With regard to the rapid progression of MLD, also in later onset forms, once independent walking is lost [3, 5], we emphasize the need to “plan ahead” when providing assistive devices. We also could show that the absolute number of devices used decreases in the late stage of the disease, when the patients could no longer use certain aids at all (such as devices for mobility or devices for verticalization such as sitting or standing). There seems to be a “maximum” of devices needed before the final stage of the disease. In a recent study on 100 patients with PMD, authors similarly describe the use of devices getting less frequent over time [41].

Such detailed data on medical aids during disease course in MLD as in the study presented here have not yet been reported in the literature. As the burden of assistive devices reflects the burden of disease in general, a reduced number of medical aids, and also the use of more “active” devices, might in future be a measure for patients’ benefit from therapy. This is illustrated in a recent report of a 6-year-old girl with LI MLD after gene therapy [16] when comparing her use of only single active medical aids with the amount of devices used by the untreated children in our study.

**Supportive therapies** like occupational, physical and speech therapy are recommended for leukodystrophy patients in general [26]. Effects are described for example for spasticity management, prevention of scoliosis [26], or to preserve motor skills [36]. Physiotherapists play a role in assessing “sensorimotor function, eating and swallowing” and help to ameliorate mobilization and also suggest and check aids [36]. In addition, therapists may assess daily activities in post-treatment outcome studies, for example by physical activity scales [46]. Physiotherapy was the most commonly applied therapy in general in this study. It can be regarded as the most important therapy right from the beginning for patients with motor onset and for all patients in advanced stages of MLD. In contrast, in a study on PMD patients, authors report on physical exercise in only 77% of the patients [41]. Occupational therapy, early intervention and speech-therapy were less reported after the period of regression. With regard to the time resources of therapy, the therapy-related burden on families becomes clear.

There was the need for **special education** in patients of this study in advanced disease stage 3. The learning environment in the FC was characterized by the need for a support staff in kindergarten/school, special bus transport, the change from regular to special education, or even the dropping-out of any educational system. None of the follow-up patients finished regular school. This fits to what is reported by Thomas et al. for the UK/Ireland (10 LI and 3 EJ MLD patients) [32]. It is consensus that in progressive disorders, school environment has to be carefully “reassessed and adjusted to the child’s evolving needs” [36]. Detailed description about learning environment in patients with MLD or other leukodystrophies in literature is scarce.

As special education consumes significant public resources, it is worth noting that even day-to-day care for children in special-needs institutions did not prevent parents from quitting their jobs, because their work was no longer compatible with caring for the severely affected child.

Patients of the FC required broad **sociomedical support** with all relevant items for severe disability on the ID card. They also received the highest level of care and the highest financial support in the healthcare system. Parents addressed the need of a full-time caregiver, as described in literature [32, 36, 39]. Daily home-nursing and weekly palliative care were considered as the most helpful support from the parents’ point of view. There was a tendency that families with older children sought support from volunteers, neighborhood, and friends, while families with younger children were supported by family members. Nursery hours were comparable (on average slightly below) to what was reported in other studies [27, 30, 32]. For some families, some relevant costs of care and equipment were not covered by the healthcare system. Even more impact had the financial loss due to extended parental leave or because parents had to quit their jobs to care for their sick child. Other authors also described a degradation in employment status because of a child’s illness [27, 31, 32, 47, 48]. (This point becomes particularly relevant when the situation persists over a long period of time or when caring for a child of a late-onset MLD form in an advanced disease stage.)

Only a quarter of the affected children in the FC “actively” participated in **family life** (e. g. were fed on family meals), but most children retained a "passive" participation (e. g., were present at family meals, but were fed afterwards), and only a few did no longer participate in family life (e. g. were fed independently of family meals or activity by nursing staff). Children in an advanced disease stage could no longer communicate verbally, but communicated in a non-verbal manner through pointing, facial expressions or gestures, or even "indirectly" via pain expression or with vegetative reactions. The difference between complete absence of communication and the preserved ability to communicate “yes” or “no” has been shown to be critical for quality of life [30, 36]. Only half of the children had social contacts beyond family life and school, for example with neighbors or friends. In other studies, parents and other family members also reported limitations in their social activities [32, 47, 48]. Nearly 75% of families indicated “fear of the future” as most burdensome, while the items “seeing the child suffer”, “daily workload” or “nursing” played a minor role. These data reflect the burden of the disease from an individual perspective of parents and families, beyond what is described for healthcare utilization.

## Conclusion

Diagnostic procedures and routine therapeutic pathways in MLD as well as sociomedical support require intensive healthcare resources. Our data illustrate the extent of hospitalization, home/palliative-care, and socio-medical support in children with late infantile and juvenile MLD in Germany. We also give detailed insight into MLD-related drugs, devices, supportive therapies, and learning environment. These data describe the high burden of MLD for patients and their families and will help to analyze families’ needs. They may also serve as a reference for the evaluation of disease- and healthcare burden after gene-/stem cell-therapy.

## Supplementary Information

Additional file 1

## Data Availability

The datasets during and/or analysed during the current study available from the corresponding author on reasonable request. All data generated or analysed during this study are included in this published article [and its supplementary information files].

## Abbreviations

AFO: Ancle foot orthosis
cMRI: Cerebral magnetic resonance imaging
DI: Date of interview
DQ: Date of Questionnaire
EJ: Early-juvenile
ERT: Enzyme replacement therapy
FC: Follow-up cohort
GMFC-MLD: Gross Motor function in MLD
ITB: Intrathecal baclofen
LI: Late infantile
LJ: Late-juvenile
MLD: Metachromatic leukodystrophy
NCV: Nerve conduction velocity
PMD: Pelizaeus-Merzbacher Disease
PEG: Percutaneous endoscopic gastro-tube
TC: Total cohort

## References

[1] Gieselmann V, Krägeloh-Mann I. Metachromatic leukodystrophy—an update. Neuropediatrics. 2010;41:1–6. 20571983 10.1055/s-0030-1253412

[2] von Figura K, Gieselmann V, Jaeken J. Metachromatic leukodystrophy. In: Scriver CR, Beaudet AL, Sly WS, Valle D, editors. The metabolic and molecular bases of inherited disease. New York: McGraw-Hill; 2001. p. 3695–724.

[3] Kehrer C, Blumenstock G, Gieselmann V, Krägeloh-Mann I, GERMAN LEUKONET. The natural course of gross motor deterioration in metachromatic leukodystrophy. Dev Med Child Neurol. 2011;53:850–5. 21707604 10.1111/j.1469-8749.2011.04028.x

[4] Kehrer C, Groeschel S, Kustermann-Kuhn B, et al. Language and cognition in children with metachromatic leukodystrophy: onset and natural course in a nationwide cohort. Orphanet J Rare Dis. 2014;9:18. 24499656 10.1186/1750-1172-9-18 PMC3922034

[5] Kehrer C, Elgün S, Raabe C, et al. Association of age at onset and first symptoms with disease progression in patients with metachromatic leukodystrophy. Neurology. 2021;96:e255–66. 33046606 10.1212/WNL.0000000000011047

[6] Fumagalli F, Zambon AA, Rancoita PMV, et al. Metachromatic leukodystrophy: a single-center longitudinal study of 45 patients. J Inherit Metab Dis. 2021;44:1151–64. 33855715 10.1002/jimd.12388

[7] Batzios SP, Zafeiriou DI. Developing treatment options for metachromatic leukodystrophy. Mol Genet Metab. 2012;105:56–63. 22078456 10.1016/j.ymgme.2011.10.002

[8] Van Egmond ME, Boelens JJ, et al. Improvement of white matter changes on neuroimaging modalities after stem cell transplant in metachromatic leukodystrophy. JAMA Neurol. 2013;70:779–82. 23608771 10.1001/jamaneurol.2013.629

[9] Groeschel S, Kühl JS, Bley AE, et al. Long-term outcome of allogeneic hematopoietic stem cell transplantation in patients with juvenile metachromatic leukodystrophy compared with nontransplanted control patients. JAMA Neurol. 2016;73:1133–40. 27400410 10.1001/jamaneurol.2016.2067

[10] Boucher AA, Miller W, Shanley R, et al. Long-term outcomes after allogeneic hematopoietic stem cell transplantation for metachromatic leukodystrophy: the largest single-institution cohort report. Orphanet J Rare Dis. 2015;10:94. 26245762 10.1186/s13023-015-0313-y PMC4545855

[11] Van Rappard DF, Boelens JJ, van Egmond ME, et al. Efficacy of hematopoietic cell transplantation in metachromatic leukodystrophy: the Dutch experience. Blood. 2016;127:3098–101. 27118454 10.1182/blood-2016-03-708479

[12] Krägeloh-Mann I, Groeschel S, Kehrer C, et al. Juvenile metachromatic leukodystrophy 10 years posttransplant compared with a non-transplanted cohort. Bone Marrow Transplant. 2013;48:369–75. 22941383 10.1038/bmt.2012.155

[13] Sessa M, Lorioli L, Fumagalli F, et al. Lentiviral haemopoietic stem-cell gene therapy in early-onset metachromatic leukodystrophy: an ad-hoc analysis of a non-randomised, open-label, phase 1/2 trial. Lancet. 2016;388:476–87. 27289174 10.1016/S0140-6736(16)30374-9

[14] Biffi A, Montini E, Lorioli L, et al. Lentiviral hematopoietic stem cell gene therapy benefits metachromatic leukodystrophy. Science. 2013;341:1233158. 23845948 10.1126/science.1233158

[15] Fumagalli F, Calbi V, Sora MGN, et al. Lentiviral haematopoietic stem-cell gene therapy for early-onset metachromatic leukodystrophy: long-term results from a non-randomised, open-label, phase 1/2 trial and expanded access. Lancet. 2022;399:372–83. 35065785 10.1016/S0140-6736(21)02017-1 PMC8795071

[16] Faccioli S, Sassi S, Pandarese D, et al. Preserving ambulation in a gene therapy-treated girl affected by metachromatic leukodystrophy: a case report. J Pers Med. 2023;13:637. 37109023 10.3390/jpm13040637 PMC10144348

[17] 2020. Libmeldy – EPAR Medicine Overview. https://www.ema.europa.eu/en/documents/overview/libmeldy-epar-medicine-overview_en.pdf

[18] Morton G, Thomas S, Roberts P, Clark V, Imrie J, Morrison A. The importance of early diagnosis and views on newborn screening in metachromatic leukodystrophy: results of a Caregiver Survey in the UK and Republic of Ireland. Orphanet J Rare Dis. 2022;17:403. 36329444 10.1186/s13023-022-02550-z PMC9635117

[19] Hong X, Daiker J, Sadilek M, et al. Toward newborn screening of metachromatic leukodystrophy: results from analysis of over 27,000 newborn dried blood spots. Genet Med. 2021;23:555–61. 33214709 10.1038/s41436-020-01017-5 PMC10395749

[20] Bonkowski JL, Nelson C, Kingston JL, Filloux FM, Mundorff MB, Srivastava R. The burden of inherited leukodystrophies in children. Neurology. 2010;75:718–25. 20660364 10.1212/WNL.0b013e3181eee46b PMC2931652

[21] Nelson C, Mundorff MB, Korgenski EK, Brimley CJ, Srivastava R, Bonkowski JL. Determinants of health care use in a population-based leukodystrophy cohort. J Pediatr. 2013;162:624–8. 23069195 10.1016/j.jpeds.2012.08.046 PMC3549018

[22] Brimley CJ, Lopez J, van Haren K, et al. National variation in costs and mortality for Leukodystrophy patients in US children’s hospitals. Pediatr Neurol. 2013;49:156–62. 23953952 10.1016/j.pediatrneurol.2013.06.006 PMC3748620

[23] Richards J, Korgenski EK, Srivastava R, Bonkowsky JL. Costs of the diagnostic odyssey in children with inherited leukodystrophies. Neurology. 2015;85:1167–70. 26320197 10.1212/WNL.0000000000001974 PMC4603881

[24] Bonkowski JL, Wilkes J, Shyr DC. Scope and burden of non-standard of care hematopoietic stem cell transplantation in pediatric leukodystrophy patients. J Child Neurol. 2018;33:882–7. 30261790 10.1177/0883073818798090

[25] Ghabash G, Wilkes J, Berney BJ, et al. Hospitalization burden and incidence of Krabbe disease. J Child Neurol. 2022;37:12–9. 34670440 10.1177/08830738211027717 PMC8671150

[26] Bonkowsky JL, Keller S. Leukodystrophies in children: diagnosis, care, and treatment. AAP section on neurology, council on genetics. Pediatrics. 2021;148:e2021053126. 34426533 10.1542/peds.2021-053126

[27] Eichler FS, Cox TM, Crombez E, í Dali C, Kohlschütter A. Metachromatic leukodystrophy: an assessment of disease burden. J Child Neurol. 2016;31:1457–63. 27389394 10.1177/0883073816656401

[28] Harrington M, Whalley D, Twiss J, et al. Insights into the natural history of metachromatic leukodystrophy from interviews with caregivers. Orphanet J Rare Dis. 2019;14:89. 31036045 10.1186/s13023-019-1060-2 PMC6489348

[29] Eichler F, Sevin C, Barth M, et al. Understanding caregiver descriptions of initial signs and symptoms to improve diagnosis of metachromatic leukodystrophy. Orphanet J Rare Dis. 2022;17:370. 36195888 10.1186/s13023-022-02518-z PMC9531467

[30] Ammann-Schnell L, Groeschel S, Kehrer C, Frölich S, Krägeloh-Mann I. The impact of severe rare chronic neurological disease in childhood on the quality of life of families: a study on MLD and PCH2. Orphanet J Rare Dis. 2021;16:211. 33971942 10.1186/s13023-021-01828-y PMC8111977

[31] Sevin C, Barth M, Wilds A, et al. An international study of caregiver-reported burden and quality of life in metachromatic leukodystrophy. Orphanet J Rare Dis. 2022;17:329. 36056437 10.1186/s13023-022-02501-8 PMC9438185

[32] Thomas S, Morrison A, Morton G, et al. The burden of disease in metachromatic leukodystrophy: results of a caregiver survey in the UK and Republic of Ireland. Orphanet J Rare Dis. 2024;19:87. 38403596 10.1186/s13023-023-03001-z PMC10895743

[33] Kehrer C, Blumenstock G, Raabe C, Krägeloh-Mann I. Development and reliability of a classification system for gross motor function in children with metachromatic leucodystrophy. Dev Med Child Neurol. 2011;53:156–60. 21087233 10.1111/j.1469-8749.2010.03821.x

[34] Ashrafi MR, Amant M, Garshabi M, et al. An update on clinical, pathological, diagnostic, and therapeutic perspectives of childhood leukodystrophies. Expert Rev Neurother. 2020;20:65–84. 31829048 10.1080/14737175.2020.1699060

[35] Van der Knaap MS, Schiffmann R, Mochel F, et al. Diagnosis, prognosis, and treatment of leukodystrophies. Lancet Neurol. 2019;18:962–72. 31307818 10.1016/S1474-4422(19)30143-7

[36] Adang LA, Sherbini O, Ball L, et al. Global Leukodystrophy Initiative (GLIA) Consortium. Revised consensus statement on the preventive and symptomatic care of patients with leukodystrophies. Mol Genet Metab. 2017;122:18–32. 28863857 10.1016/j.ymgme.2017.08.006 PMC8018711

[37] Chang SC, Eichinger CS, Field P. The natural history and burden of illness of metachromatic leukodystrophy: a systematic literature review. Eur J Med Res. 2024;29:181. 38494502 10.1186/s40001-024-01771-1 PMC10946116

[38] Zhang J, Ban T, Zhou L, et al. Epilepsy on children with leukodystrophies. J Neurol. 2020;267:2612–8. 32388833 10.1007/s00415-020-09889-y

[39] Van Haren K, Bonkowsky JL, Bernard G, et al. Consensus statement on preventive and symptomatic care of leukodystrophy patients. Mol Genet Metab. 2015;114:516–26. 25577286 10.1016/j.ymgme.2014.12.433

[40] van Rappard DF, Bugiani M, Boelens JJ, et al. Gallbladder and the risk of polyps and carcinoma in metachromatic leukodystrophy. Neurology. 2016;87:103–11. 27261095 10.1212/WNL.0000000000002811

[41] Moore KM, Wolf NI, Hobson G, et al. Pelizaeus-Merzbacher-disease: a caregiver assessment of disease impact. J Child Neurol. 2023;38:78–84. 36744386 10.1177/08830738231152658

[42] Schoenmakers DH, Beerepoot S, van den Berg S, et al. Modified Delphi procedure-based expert consensus on endpoints for an international disease registry for Metachromatic Leukodystrophy: the European Metachromatic Leukodystrophy initiative (MLDi). Orphanet J Rare Dis. 2022;17:48. 35164810 10.1186/s13023-022-02189-w PMC8842918

[43] Van der Veldt N, van Rappard DF, van de Pol LA, et al. Intrathecal baclofen in metachromatic leukodystrophy. Dev Med Child Neurol. 2019;61:232–5. 29806077 10.1111/dmcn.13919 PMC7379712

[44] Koto Y, Ueki S, Yamakawa M, et al. Experiences of patients with lysosomal storage disorders who are receiving enzyme-replacement therapy and the experiences of their family members: a qualitative systematic review. JBI Evid Synth. 2022;20:1474–510. 34839313 10.11124/JBIES-21-00074

[45] Mirchi A, Pelletier F, Tran LT, et al. Health-related quality of life for patients with genetically determined leukoencephalopathy. Pediatr Neurol. 2018;84:21–6. 29859719 10.1016/j.pediatrneurol.2018.03.015

[46] Brown M, Martin S, Fehnel SE, Deal LS. Development of the impact of juvenile metachromatic leukodystrophy on physical activities scale. J Patient Rep Outcomes. 2018;2:15. 10.1186/s41687-018-0041-x PMC5934920 29757307

[47] Harrington M, Hareendran A, Skalicky A, et al. Assessing the impact on caregivers caring for patients with rare pediatric lysosomal storage disease: development of the Caregiver Impact Questionnaire. J Patient Rep Outcomes. 2019;3:44. 31338630 10.1186/s41687-019-0140-3 PMC6650510

[48] Koto Y, Yamashita W, Sakai N. Impact on physical, social, and family functioning of patients with metachromatic leukodystrophy and their family members in Japan: a qualitative study. Mol Genet Metab Rep. 2024;38: 101059. 38469094 10.1016/j.ymgmr.2024.101059 PMC10926226

