# Supplementary material for: Healthcare utilization and disease burden in children with metachromatic leukodystrophy in Germany
Source: Orphanet J Rare Dis. 2025 May 23;20:242. doi: 10.1186/s13023-025-03637-z (PMC12100800; doi:10.1186/s13023-025-03637-z)
Supplement: Supplementary file 1 — Additional file 1 [file 13023_2025_3637_MOESM1_ESM.docx]

Supplementary Table 1

1. Drug use of the total cohort (n = 83)
   n = number of patients using a respective drug

| Analgesic (n = 22) | gabapentin | n=5 |
| --- | --- | --- |
|  | ibuprofen | n=6 |
|  | metamizole | n=2 |
|  | morphine | n=2 |
|  | paracetamol | n=3 |
|  | pregabalin | n=2 |
|  | tilidine | n=1 |
|  | tramadol | n=1 |
| Spasmolytic (n = 45) | baclofen | n=19 |
|  | botulinum toxin | n=1 |
|  | L-dopa | n=1 |
|  | tetrazepam | n=3 |
|  | tizanidine | n=3 |
|  | vigabatrin | n=18 |
| Sedative (n = 24) | chloral hydrate | n=5 |
|  | diazepam | n=4 |
|  | dronabinol | n=2 |
|  | lorazepam | n=1 |
|  | melatonin | n=4 |
|  | nitrazepam | n=5 |
|  | opipramol | n=1 |
|  | oxazepam | n=1 |
|  | promethazine | n=1 |
| Anticonvulsant (n = 15) | carbamazepine | n=1 |
|  | clobazam | n=3 |
|  | clonazepam | n=2 |
|  | lamotrigine | n=1 |
|  | levetiracetam | n=2 |
|  | oxcarbazepine | n=1 |
|  | phenobarbital | n=1 |
|  | topiramate | n=2 |
|  | valproate | n=2 |
| Gastrointestinal drug (n = 24) | esomeprazole | n=1 |
|  | lactulose | n=3 |
|  | sorbitol | n=1 |
|  | omeprazole | n=7 |
|  | polyethylene glycol | n=8 |
|  | scopolamine | n=1 |
|  | trospium chloride | n=1 |
|  | ursodeoxycholic acid | n=2 |
| Antibiotic (n = 2) | nitrofurantin | n=2 |
| Inhalant  (n = 4) | acetylcysteine | n=2 |
|  | guaifenesin | n=1 |
|  | salbutamol | n=1 |
| Dietary supplement (n = 7) | CDP-choline | n=1 |
|  | coenzyme Q10 | n=1 |
|  | iron | n=1 |
|  | vitamin B | n=1 |
|  | vitamin D | n=3 |
| Others (n = 3) | amitriptyline | n=1 |
|  | gingko | n=1 |
|  | hydrochlorothiazide | n=1 |

1. Drug use of the follow-up cohort (n = 19)
   n = number of patients using a respective drug

| Analgesic | gabapentin | n=2 |
| --- | --- | --- |
| (n = 10) | metamizole | n=2 |
|  | morphine | n=2 |
|  | pregabalin | n=4 |
| Spasmolytic | baclofen | n=13 |
| (n = 20) | vigabatrin | n=7 |
| Sedative | diazepam | n=4 |
| (n = 14) | melatonin | n=4 |
|  | nitrazepam | n=2 |
|  | tetrahydrocannabinol | n=4 |
| Anticonvulsant | clobazam | n=3 |
| (n = 14) | clonazepam | n=2 |
|  | lacosamide | n=1 |
|  | levetiracetam | n=3 |
|  | midazolam | n=1 |
|  | phenobarbital | n=2 |
|  | valproate | n=1 |
|  | zonisamide | n=1 |
| Gastrointestinal drug | D-mannose | n=1 |
| (n = 32) | domperidone | n=3 |
|  | maltodextrin | n=1 |
|  | naloxegol | n=1 |
|  | omeprazole | n=10 |
|  | polyethylene glycol | n=7 |
|  | scopolamine | n=2 |
|  | sorbitol | n=1 |
|  | ursodeoxycholic acid | n=6 |
| Antibiotic | nitrofurantin | n=2 |
| (n = 3) | trimethoprim | n=1 |
| Inhalant | budesonide | n=1 |
| (n = 8) | glycopyrronium bromide | n=2 |
|  | ipratropium bromide | n=1 |
|  | NaCl | n=2 |
|  | salbutamol | n=2 |
| Dietary supplement | chromium | n=1 |
| (n = 18) | magnesium | n=1 |
|  | Omega 3 | n=1 |
|  | potassium | n=2 |
|  | probiotics | n=1 |
|  | Q10 | n=1 |
|  | Schüssler salts | n=1 |
|  | selenium | n=2 |
|  | vitamin C | n=1 |
|  | vitamin D | n=5 |
|  | zinc | n=2 |
| Others | amitriptyline | n=1 |
| (n = 2) | eslicarbazepine acetate | n=1 |
